# Diversifying selection drives parallel evolution of gill raker number and body size along the speciation continuum of European whitefish

**DOI:** 10.1002/ece3.3876

**Published:** 2018-02-05

**Authors:** Katja Häkli, Kjartan Østbye, Kimmo K. Kahilainen, Per‐Arne Amundsen, Kim Præbel

**Affiliations:** ^1^ Faculty of Biosciences, Fisheries and Economics Norwegian College of Fishery Science UiT The Arctic University of Norway Tromsø Norway; ^2^ Faculty of Applied Ecology and Agricultural Sciences Hedmark University of Applied Science Elverum Norway; ^3^ Department of Biosciences Centre for Ecological and Evolutionary Synthesis (CEES) University of Oslo Oslo Norway; ^4^ Department of Arctic and Marine Biology Faculty of Biosciences, Fisheries and Economics UiT The Arctic University of Norway Tromsø Norway

**Keywords:** adaptation, *Coregonus lavaretus*, drift, gill rakers, phenotype‐environment correlation, total length

## Abstract

Adaptive radiation is the evolution of ecological and phenotypical diversity. It arises via ecological opportunity that promotes the exploration of underutilized or novel niches mediating specialization and reproductive isolation. The assumed precondition for rapid local adaptation is diversifying natural selection, but random genetic drift could also be a major driver of this process. We used 27 populations of European whitefish (*Coregonus lavaretus*) from nine lakes distributed in three neighboring subarctic watercourses in northern Fennoscandia as a model to test the importance of random drift versus diversifying natural selection for parallel evolution of adaptive phenotypic traits. We contrasted variation for two key adaptive phenotypic traits correlated with resource utilization of polymorphic fish; the number of gill rakers and the total length of fish, with the posterior distribution of neutral genetic differentiation from 13 microsatellite loci, to test whether the observed phenotypic divergence could be achieved by random genetic drift alone. Our results show that both traits have been under diversifying selection and that the evolution of these morphs has been driven by isolation through habitat adaptations. We conclude that diversifying selection acting on gill raker number and body size has played a significant role in the ongoing adaptive radiation of European whitefish morphs in this region.

## 1 INTRODUCTION

Adaptive radiation is a process where a lineage diversifies into new lineages adapted to divergent environments, which results in phenotype‐environment associations, niche specialization, and genetic divergence (Gavrilets & Vose, 2005). Natural selection acts as a driving force for rapid local adaptation (Barrett & Schluter, 2008), whereas random genetic drift, mutations, and gene flow may play a role in changing gene frequencies in populations. Notwithstanding, nonadaptive processes, such as genetic linkage and pleiotropy of traits, may also play important roles in the phenotypic and genotypic differentiation involved in adaptive radiations (Schluter, 2000). The genetic architecture of traits, together with the adaptive genetic variation, upon which various selection pressures are exerted, will set the frame for the response to natural selection (Nosil, Funk, & Ortiz‐Barrientos, 2009). However, divergence of populations, and eventually speciation, can also occur via random genetic drift (Lee, Shaner, Lin, & Lin, 2016; Uyeda, Arnold, Hohenlohe, & Mead, 2009). Because of this complexity, the mechanisms by which evolution modulates phenotypic and genotypic frequencies in the divergence process are not well understood. In order to detect selection and rule out other causes as an explanation for the differentiation of phenotypic traits, it is necessary to compare the observed differentiation with neutral differentiation expected under random genetic drift. Although this has been attempted by contrasting neutral genetic (*F*_ST_) and quantitative trait (*Q*_ST_) differentiation (Brommer, 2011; Østbye, Næsje, Bernatchez, Sandlund, & Hindar, 2005; Ozerov et al., 2015; Whitlock, 2008), assessing the interplay between environmental and genetic causes of differentiation has been problematic as *F*_ST_/*Q*_ST_ comparison does not allow for detection of interactions between phenotypes, genotypes, and the environment (Pujol, Wilson, Ross, & Pannell, 2008). Therefore, it is often challenging to disentangle whether the observed differentiation in phenotypic traits is a response to natural selection or simply just a plastic response to environmental differences, especially when the number of populations is small and they are subject to strong random genetic drift (Brommer, 2011; Leinonen, McCairns, O'Hara, & Merilä, 2013; Ovaskainen, Karhunen, Zheng, Arias, & Merilä, 2011; Pujol et al., 2008). However, recent efforts in coupling quantitative and population genetic theory have created realistic models (Ovaskainen et al., 2011) and tools (R package “driftsel,” Karhunen, Merilä, Leinonen, Cano, & Ovaskainen, 2013), for this exercise. Using the Bayesian methods implemented in “driftsel,” it is now possible to contrast and statistically test differentiation of phenotypic traits under scenarios of random genetic drift and diversifying selection, and thereby compare possible similarities among phenotypes and environments (e.g., habitats) even with small number of populations or when *Q*_ST_ equals *F*_ST_ (Ovaskainen et al., 2011).

The numerous postglacial lakes harboring polymorphic fish populations in Fennoscandia are relatively young (<10 kyr) and represent discrete and isolated environments, making them outstanding “natural laboratories” for studying processes that initiate and maintain niche adaptation and population divergence. European whitefish (*Coregonus lavaretus* [L.]) is a highly abundant fish species in these lakes and has diverged into distinct morphs adapted to the three principal lake habitats (littoral, pelagic, and profundal; Harrod, Mallela, & Kahilainen, 2010; Kahilainen & Østbye, 2006; Præbel, Knudsen, et al., 2013; Siwertsson et al., 2010). The morphs are discriminated based on the head morphology and the number of gill rakers (Amundsen, Bøhn, & Våga, 2004; Kahilainen & Østbye, 2006; Siwertsson et al., 2010). In addition, the morphs differ in body size, where the large‐bodied individuals are found in the most profitable foraging habitat, the littoral zone, whereas smaller individuals are found in the pelagic and profundal zones (Bøhn & Amundsen, 2004; Kahilainen, Alajärvi, & Lehtonen, 2005; Kahilainen, Lehtonen, & Könönen, 2003). The number of gill rakers is a heritable and ecologically important trait (Bernatchez, 2004; Svärdson, 1952, 1979), associated with diet preference (Østbye et al., 2006; Siwertsson, Knudsen, Adams, Præbel, & Amundsen, 2013). The whitefish morphs are named according to their body size and the number of gill rakers (Kahilainen & Østbye, 2006) as follows: large sparsely rakered (LSR) whitefish with intermediate number of gill rakers, densely rakered (DR) whitefish with the highest number of gill rakers, and small sparsely rakered (SSR) whitefish with the lowest number of gill rakers. LSR whitefish mainly feeds on benthic macroinvertebrates in the littoral habitat, DR whitefish is a zooplanktivorous specialist that resides in the pelagic habitat, and SSR is a specialized benthivore consuming profundal benthic invertebrates (Harrod et al., 2010).

Previous studies have suggested that the variation in the degree of divergence among the morphs throughout northern Fennoscandia represents a speciation continuum within watercourses and at a broader landscape level (Østbye, Næsje, et al., 2005; Østbye et al., 2006; Siwertsson et al., 2010). The ubiquitous LSR whitefish has been regarded as the ancestral phenotype from which the other morphs have evolved (Østbye, Bernatchez, Næsje, Himberg, & Hindar, 2005), as this morph is present in all lakes and the only morph found in allopatry. The most diversified systems in this region are found close to the main stem in the easternmost Pasvik watercourse (Siwertsson et al., 2010), where most lakes harbor all three whitefish morphs (DR, LSR, and SSR; Harrod et al., 2010; Kahilainen & Østbye, 2006; Præbel, Knudsen, et al., 2013). The same pattern of the main stem lakes being the most diversified also holds true for the more western watercourses, despite that the radiations are less developed. In the Tana and Alta watercourses, all three morphs are phenotypically recognized, but the SSR morph appears to be genetically less diverged from the LSR morph compared to the radiation in Pasvik (Præbel, Knudsen, et al., 2013; Siwertsson et al., 2010; Siwertsson, Knudsen, Præbel, et al., 2013). Intraspecific and interspecific genetic diversity of the whitefish morphs also decreases from the Pasvik to the Alta watercourse, so that the allelic richness in the Tana and Alta watercourses is just a subset of the allelic richness found in the Pasvik watercourse (Østbye et al., 2006). The reduced allelic richness in the Tana and Alta watercourses has been hypothesized to be related to the postglacial colonization route from east to west by a single clade of whitefish (Østbye, Bernatchez, et al., 2005). The postglacial colonization likely followed the retreating ice sheet edge from east to west about 10,000 years B.P. (Andersen & Borns, 1994; Kujansuu, Eriksson, & Grönlund, 1998; Mangerud et al., 2004; Sollid et al., 1973; Svendsen et al., 2004). As a consequence, the whitefish populations in the Pasvik watercourse are expectedly 5,000 years older compared to the whitefish populations in the Alta watercourse (Præbel, Knudsen, et al., 2013).

There is a considerable amount of ecological studies that suggest natural selection as a main cause behind divergent whitefish populations (e.g., Amundsen et al., 2004; Lu & Bernatchez, 1999; Siwertsson et al., 2010), but only a few studies have attempted to test whether phenotypic traits, gill raker number and body size have an adaptive role in the divergence process in whitefish (Østbye, Næsje, et al., 2005; Præbel, Knudsen, et al., 2013; Rogers, Gagnon, & Bernatchez, 2002; Vonlanthen et al., 2009). Divergence in gill raker and body size traits is commonly detected in a range of different postglacial fish species along speciation continuums, suggesting their key importance toward increasing specialization into pelagic or benthic niches (Hendry et al. 2009). Previous phenotypic‐genotypic variation comparisons of gill raker counts in pelagic and benthic lake whitefish (*Coregonus clupeaformis*) and European whitefish have revealed deviation from neutral expectation, suggesting that the number of gill rakers has evolved as a product of natural selection (Østbye, Næsje, et al., 2005; Præbel, Knudsen, et al., 2013; Rogers et al., 2002). However, these previous studies have focused on single or a few neighboring lakes, whereas landscape level approaches are missing. Range expansion of species to new areas generally leads to reduced allelic richness and heterozygosity (Besold, Schmitt, Tammaru, & Cassel‐Lundhagen, 2008; White, Perkins, Heckel, & Searle, 2013). These repeated founder events build up genetic differentiation through a spatial analog of genetic drift (Slatkin & Excoffier, 2012). In the case of northern Fennoscandian whitefish, the range expansion from the oldest Pasvik watercourse populations toward the progressively younger Tana and Alta watercourse populations likely conserve the footprints of the colonization history as, for example, manifested in a diminishing amount of genetic variation. However, whether evolution of similar adaptive phenotypes in different lakes and watercourses with repeated founder events are driven by diversifying selection or originate from random genetic drift, remains to be tested.

This study assesses the different stages of divergence along the speciation continuum for all three whitefish morphs throughout the wide northern Fennoscandian landscape. Our objectives were to investigate i) whether diversifying selection or the repeated events of genetic drift from the postglacial recolonization has shaped phenotypic traits of whitefish into three different adaptive modes in the three lake habitats, and ii) whether the habitat is more important for the observed patterns of natural selection than lakes or watercourses, thus reflecting the action of parallel evolution. If random genetic drift is the main driver of the phenotypic divergence, we expect to observe random genotypic and phenotypic clustering of morphs within lakes and watercourses.

## 2 MATERIALS AND METHODS

**Figure 1:**
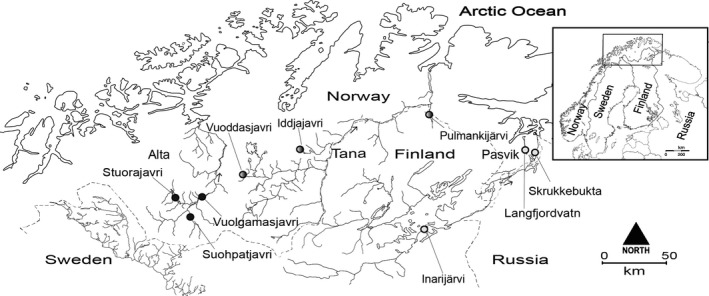
Map of northern Europe and close up of the northern Fennoscandian location of the study sites. All three watercourses are well separated and drain to different fjords in the Arctic Ocean. Open dots indicate study lakes in the Pasvik watercourse, gray dots the Tana watercourse, and black dots the Alta watercourse. For Inarijärvi, the dot is located to the specific sampling site within the lake

We collected whitefish in nine lakes from three subarctic watercourses: Suohpatjavri, Stuorajavri, and Vuolgamasjavri from Alta watercourse, Iddjajavri, Vuoddasjavri, and Pulmankijärvi from Tana watercourse, and Inarijärvi, Skrukkebukta, and Langfjordvatn from Pasvik watercourse (Figure 1). We chose large (2–32 km^2^) and deep (max 25–53 m) oligotrophic lakes where all the three principal habitats (littoral, pelagic, and profundal) were present. Fish were sampled from all habitats using benthic and pelagic gill net series (mesh sizes 10–60 mm). The fish were removed from gill nets and visually classified to morph according to appearance, head, and body form, and by their gill raker morphology as described by Kahilainen and Østbye (2006). The total length (accuracy 1 mm) was measured; fin clip or a piece of gill filament was taken for genetic analyses, followed by visual inspection of gonads for sex determination and assessment of sexual maturity. The number of gill rakers was counted under a microscope on the first left branchial arch to verify the right morph assignment. Individuals with undefined morph record or morphs caught in a non‐native habitat were excluded. Without physical boundaries between different lake habitats, the morphs are not restricted only to their native habitat. However, analyses of stomach contents have uncovered different diet preferences and low niche overlap between the three distinct whitefish morphs (Harrod et al., 2010; Kahilainen & Østbye, 2006; Østbye et al., 2006). These criteria lead to a dataset with a total number of 999 individuals, from which we had the following information: morph classification, sex, maturity, number of gill rakers, total length, and data from 21 microsatellite loci.

Stable isotope analyses in trimorphic lakes in the Pasvik watercourse have showed differences in resource use between morphs (Harrod et al., 2010; Præbel, Knudsen, et al., 2013). Despite the less prominent divergence between LSR and SSR whitefish in the Alta watercourse, the stable isotope ratios of carbon and nitrogen have also there revealed two ecological clusters, the profundal and the littoral, within the benthic whitefish, demonstrating that the capture habitat of the benthic fish is a good indicator of long‐term resource use (Siwertsson, Knudsen, Præbel, et al., 2013). We assume that the same association holds true for the Tana watercourse—logically also—due to the colonization history and its location between the Pasvik and Alta watercourses. In addition to the habitat and dietary segregation, littoral and profundal whitefish in the Alta watercourse differ in head morphology (Siwertsson, Knudsen, Adams, et al., 2013). Further, small but statistically significant genetic differences have also been observed between littoral and profundal whitefish (Siwertsson, Knudsen, Præbel, et al., 2013).

The number of gill rakers in the different morphs represented three partly overlapping unimodal distributions where SSR had gill raker number from 18 to 24, LSR from 22 to 39, and DR from 27 to 41. SSR whitefish has shorter gill rakers, robust head, and larger eyes compared to LSR whitefish, whereas DR has long gill rakers and pointed head shape (Harrod et al., 2010; Kahilainen & Østbye, 2006). In order to study the populations at different positions along the speciation continuum, whitefish caught in the profundal habitat with gill raker counts 28 or lower, were coded as SSR whitefish in Tana and Alta watercourses (but see also Siwertsson et al., 2010; Siwertsson, Knudsen, Adams, et al., 2013; Siwertsson, Knudsen, Præbel, et al., 2013). As the SSR whitefish in the Tana and Alta watercourses are less diverged from the LSR whitefish than in the Pasvik watercourse, the upper limit for the number of gill rakers was set at a higher number than what the SSR whitefish in Pasvik watercourse usually have, and thus overlapped more with LSR whitefish.

### 2.1 Phenotypic traits

We assessed the divergence among morphs with regard to two common phenotypic traits of postglacial fish radiations (Hendry, 2009; Schluter, 2000): body size (the total length of fish) and the number of gill rakers. The number of gill rakers is known to be a highly heritable trait (Svärdson, 1952, 1979), which also correlates with dietary niche and obviously shows a trait utility in terms of foraging (Kahilainen et al., 2011; Østbye et al., 2006). The correlation between parents and progeny with regard to the number of gill rakers is strong, as the heritability estimate (*h* ^2^) for the trait is high (0.79; Bernatchez, 2004; Svärdson, 1952, 1979). Heritability estimates for body length in salmonid fish vary from 0.08 to 0.42 (Gjerde & Gjedrem, 1984; Gjerde & Schaeffer, 1989; Gunnes & Gjedrem, 1978; Refstie & Steine, 1978; Standal & Gjerde, 1987). As a phenotypic trait, the body length not only represents the size of the fish but also often strongly correlates with weight, condition, age, maturity, and sex. The smaller heritability estimates for body length also mean that the effect of additive genetic variation for this trait is smaller and that environmental factors may have more effect on the phenotypic difference in total length of fish than in the number of gill rakers. Although the number of mature and immature individuals was fairly equal in our dataset (Table 1), we added maturity and sex as fixed effects in our model to account for the age of the fish and possible effects of sexual dimorphism on the phenotypic traits. Both fixed effects were treated as binary traits. The dataset included 37 individuals with unknown sex and 19 individuals with unknown maturity records (Table 1). As sex was not associated with the total length of fish or the number of gill rakers, missing data for these traits were considered not to affect the further analyses. The overall proportion of males and females in the data was 50% and 46%, respectively, while 55% of all individuals were mature (Table 1). The proportion of males in the study lakes ranged from 42% to 64%, whereas the proportion of mature fish in the samples varied from 35% to 91% among lakes (Table 1). The number of gill rakers and the total length of fish among the three morphs in all the lakes in the three watercourses were compared using analyses of variance, and multiple pairwise comparisons were performed with Tukey's HSD test in the R statistical computing programme (R Core Team, 2017).

**Table 1:** Spatial and morphometric information of the study lakes, that is, lake area, maximum depth, altitude, number of fish species present, and location of the lake

Lake	Area (km^2^)	Max depth (m)	Altitude (m a.s.l.)	No of fish species	Latitude (°N)	Longitude (°E)	*N*	Males	Females	N.A.	Mature (%)
Suohpatjavri	2.0	25	325	5	68° 56′	23° 05′	82	41	41		62
Stuorajavri	23.7	30	374	6	69° 06′	22° 49′	111	61	50		34
Vuolgamasjavri	2.8	30	301	6	69° 07′	23° 20′	114	52	62		45
Iddjajavri	6.4	30	275	5	69° 37′	25° 16′	141	74	50	17	44
Vuoddasjavri	2.9	32	334	5	69° 21′	24° 00′	142	91	51		61
Pulmankijärvi	12.0	36	12	9	70° 00′	28° 01′	123	50	72	1	59
Inarijärvi	32.0^a^	40^a^	118	13	69° 02′	27°52′	71	26	30	15	35
Skrukkebukta	6.6	37	21	8	69° 33′	30° 06′	87	44	41	2	51
Langfjordvatn	2.8	53	7	6	69° 33′	29° 57′	128	63	63	2	91

*N*, total amount of fish used in analyses; males/females, number of males/females; N.A., individuals with unknown sex; mature, proportion of mature fish on each lake.

a In Inarijärvi, sampling was confined to a single 32 km^2^ bay (Nanguvuono).

### 2.2 Microsatellite DNA/genotyping

Genomic DNA was extracted using E‐Z96 Tissue DNA Kit (OMEGA Bio‐tek) following the manufacturer's instructions. A total of 21 microsatellite loci (Table S1) were amplified in four polymerase chain reaction (PCR) multiplexes using forward‐labeled primers according to the protocol of Præbel, Westgaard, et al. (2013). The PCR products were separated on an ABI 3130 XL Automated Genetic Analyser (Applied Biosystems) using GENESCAN LIZ‐500 (Applied Biosystems) as an internal size standard. The binning and scoring were performed in GENEMAPPER 3.7 (Applied Biosystems) and manually verified. Replicate (5%–9%) and blind (4%) samples were included in all PCR's to confirm consistency of scoring and the absence of contamination. The repeatability and consistency of genotypes were 100%, and contamination was absent. The genotypes were screened for abnormalities in the software MICRO‐CHECKER 2.2.3 (Van Oosterhout, Hutchinson, Wills, & Shipley, 2004), using 1,000 bootstraps to generate the expected homozygote and heterozygote allele size difference frequencies. The microsatellite data were tested with LOSITAN (Antao, Lopes, Lopes, Beja‐Pereira, & Luikart, 2008) to obtain neutral marker data. The neutral marker data contained 13 microsatellite loci (bolded in Table S1). Deviations from Hardy–Weinberg equilibrium (HWE) and linkage disequilibrium (LD) were tested per locus over all populations using exact tests (Guo & Thompson, 1992) as implemented in GenePop 4.0 (Rousset, 2007). The pairwise comparisons were corrected for multiple comparisons using sequential Bonferroni corrections (BFC) following Rice (1989). The number of alleles at each microsatellite locus ranged from 6 to 36 across all lakes and morphs (Table S1). Deviations from HWE were indicated in 13 of 351 tests (3.7%) after sequential BFC, which are less than expected by chance (5%). None of the loci comparisons (*n* = 2,106) were significant for LD after BFC.

### 2.3 Model for genetic differentiation

We compared neutral genetic differentiation and observed quantitative genetic differentiation in order to differentiate random genetic drift from selection. Under random genetic drift, the vector of population means $a^{p}$ has the multivariate normal distribution

$$a^{p}∼N\left(\mu^{A},\;2G^{A}⊗\theta^{P}\right)$$

where $\mu^{A}$ is the common ancestral mean for all populations, $G^{A}$is the ancestral variance–covariance matrix summarizing the variances and covariance of traits, ⨂ is a Kroenecker product, an operator resulting block matrix, and $\theta^{P}$ is the population‐level coancestry matrix. The analyses were performed with RAFM (Karhunen & Ovaskainen, 2012) and Driftsel R packages (Karhunen et al., 2013). Driftsel requires two types of data, genotypic data from neutral molecular markers and quantitative data from phenotypes. The genotypic data from the 13 neutral microsatellite loci were analyzed with R package RAMF in order to obtain posterior distribution of the neutral genetic differentiation $\theta^{P}$ (coancestry coefficients). The coancestry coefficient is the summarization of the expected level of genetic similarity; in other words, it evaluates how much the individuals are expected to resemble each other. Estimation was calculated with an admixture F‐model (Karhunen & Ovaskainen, 2012) using 200,000 iterations, 50,000 burning iterations, and a thinning interval of 100. The posterior distribution from the coancestry matrix $\theta^{P}$ was then used as a prior for Driftsel to estimate the posterior distributions of other parameters and to refine the estimate of $\theta^{P}$. The function MH (a Metropolis–Hastings algorithm for quantitative genetics) in Driftsel was executed using 80,000 iterations, 40,000 burning iterations, and thinning interval of 10. Convergence of analysis was qualitatively evaluated based on visual inspection of three parallel runs. We analyzed all the three watercourses, altogether nine lakes, in one overall round, and then subsequently divided the data into separate watercourses to investigate the effect of watercourse. As Driftsel does not specify clearly which traits selection is acting on, we ran each trait separately in addition to an overall round with both traits.

The effect of physical proximity and habitat types to population structure was assessed by comparing the levels of coancestry between lakes and between habitats within each watercourse. We used the population coancestry coefficient $\theta^{P}$ to calculate the average coancestry within habitats (between lakes) and within lakes (between habitats) to investigate whether the population structure was more influenced by habitat or by lake.

In addition, we performed the formal *S* and *H* tests (Karhunen, Ovaskainen, Herczeg, & Merilä, 2014; Ovaskainen et al., 2011). Both of these tests use posterior distributions calculated with Driftsel as a prior (MH function). The *S* test evaluates how far the population means have diverged (or drifted) from the ancestral mean. It detects the signals of selection by comparing the posterior distributions of the population effects, variance–covariance matrix of the traits and the $\theta^{P}$ from neutral marker data. The *H* test measures whether population means correlate with the environmental data more than would be expected on the basis of shared evolutionary history. It includes environmental data to the neutrality test and assesses the similarities of populations found in similar habitats. The *S* and *H* tests values range from zero to one. Values close to one and zero from the *S* test imply diversifying and stabilizing selection, respectively, whereas values close to 0.5 imply perfect match with neutrality. *S* test is known to be conservative, where a *S* value of 0.95 refers to diversifying selection at 95% credibility level (Karhunen et al., 2014). A large *H* test value suggests that populations are more adapted to their environment than would be expected based on their shared phylogenetic history (Karhunen et al., 2014).

## 3 RESULTS

### 3.1 Phenotypic analysis

The number of gill rakers ranged from 15 to 47 with an overall mean of 28 ± 7.1 (±*SD*; Table 2). The mean number of gill rakers was significantly different (*p* < .001) between different morphs within all lakes in the Pasvik watercourse, where DR whitefish had the highest (34.6 ± 3.0), LSR whitefish intermediate (25.6 ± 4.7), and SSR whitefish the lowest (20.7 ± 2.7) number of gill rakers (Table 3). Also in Tana and Alta, the mean number of gill rakers was higher for DR whitefish (*p* < .001; Tana: 37.2 ± 3.4, Alta: 37.4 ± 3.5) compared to LSR (Tana: 23.4 ± 3.1, Alta: 25.7 ± 2.5) and SSR whitefish (Tana: 22.7 ± 2.1, Alta: 23.4 ± 2.6). On the contrary, when comparing the LSR and SSR whitefish, we found significant differences for number of gill rakers only in Suohpatjavri in Alta (*p* < .001) and Vuoddasjavri in Tana (*p* < .001), although in Vuolgamasjavri, the differences in number of gill rakers were close to be statistically significant (*p* = .055; Table 2). The mean total length of DR whitefish was generally smaller (18.3 ± 5.2 cm [*SD*]) than the mean total length of LSR (21.6 ± 6.5 cm) and SSR (21.3 ± 5.0 cm) whitefish (*p* < .001), but the total length of fish varied between lakes and watercourses (Tables 2 and 3). In the Pasvik watercourse, DR whitefish were smaller (12.2 ± 2.7 cm than LSR (19.4 ± 6.5 cm) whitefish, but there were no statistically significant differences between DR and SSR whitefish in Skrukkebukta. Also in Tana, the DR whitefish (18 ± 4.7 cm) were smaller in size than the other morphs (LSR: 22.2 ± 6.7 cm, SSR: 22.9 ± 5.7 cm), with a few exceptions: There were no statistically significant differences between DR and LSR whitefish in Pulmankijärvi, between DR and SSR whitefish in Iddjajavri, and between LSR and SSR whitefish in Vuoddasjavri (Table 3). In Alta, the mean lengths of all three morphs were greater than in Pasvik, but there were no significant differences in the mean total length among the morphs (DR: 22.8 ± 2.5 cm; LSR: 22.9 ± 5.7 cm; SSR: 22.2 ± 4.8 cm) in any of the three lakes.

**Table 2:** Summary table of nine study lakes indicating watercourse, lake, whitefish morph code, sample size (*N*), mean number and range of gill rakers as well as mean total length (cm) for each population

Watercourse	Lake	Morph	Code	*N*	Mean gill rakers ± *SD* (min–max)	Mean length ± *SD* (min–max)
Alta	Suohpatjavri	DR	SuD	33	40.7 ± 2.6 (37–47)	24.0 ± 1.1 (21.8–25.5)
		LSR	SuL	34	28.2 ± 2.1 (24–32)	24.4 ± 6.0 (16.5–35.5)
		SSR	SuS	15	23.7 ± 3.3 (18–28)	27.1 ± 4.0 (20.2–36.6)
	Stuorajavri	DR	StD	44	34.8 ± 2.1 (31–40)	22.0 ± 2.8 (13.1–29.5)
		LSR	StL	39	24.1 ± 1.9 (21–28)	21.0 ± 5.3 (14.0–33.7)
		SSR	StS	28	23.2 ± 2.2 (19–26)	19.7 ± 3.7 (14.0–30.1)
	Vuolgamasjavri	DR	VgD	22	37.8 ± 2.3 (33–41)	22.3 ± 2.6 (17.5–27.7)
		LSR	VgL	50	25.3 ± 1.7 (20–30)	23.5 ± 5.4 (11.5–35.1)
		SSR	VgS	42	23.3 ± 2.5 (19–28)	22.0 ± 5.4 (15.5–35.2)
Tana	Iddjajavri	DR	IdD	62	34.0 ± 2.1 (29–39)	17.2 ± 2.1 (12.6–25.0)
		LSR	IdL	56	22.1 ± 3.4 (15–34)	23.9 ± 7.5 (9.8–40.7)
		SSR	IdS	23	22.1 ± 2.0 (19–26)	17.9 ± 4.5 (11.2–28.0)
	Vuoddasjavri	DR	VdD	51	38.0 ± 2.5 (28–42)	13.0 ± 2.1 (9.3–22.1)
		LSR	VdL	48	24.2 ± 2.8 (18–31)	19.4 ± 3.7 (10.5–27.2)
		SSR	VdS	43	22.0 ± 2.1 (17–27)	20.8 ± 3.9 (10.2–33.3)
	Pulmankijärvi	DR	PuD	55	40.0 ± 2.2 (36–45)	23.5 ± 1.4 (17.1–26.2)
		LSR	PuL	31	24.2 ± 1.8 (22–28)	23.5 ± 7.7 (13.5–57.0)
		SSR	PuS	37	23.9 ± 1.6 (20–28)	28.7 ± 2.5 (20.8–32.7)
Pasvik	Inarijärvi	DR	InD	26	35.1 ± 2.6 (29–39)	11.5 ± 3.0 (6.1–17.4)
		LSR	InL	22	21.7 ± 1.4 (19–25)	26.9 ± 6.6 (18.3–46.1)
		SSR	InS	23	18.0 ± 1.4 (16–21)	21.5 ± 3.2 (13.9–25.3)
	Skrukkebukta	DR	SbD	16	33.1 ± 3.1 (29–40)	13.7 ± 3.2 (10.0–20.5)
		LSR	SbL	32	24.9 ± 2.5 (21–31)	19.9 ± 5.9 (7.7–30.4)
		SSR	SbS	39	20.0 ± 1.8 (16–23)	16.3 ± 1.9 (12.0–21.6)
	Langfjordvatn	DR	LfD	20	35.2 ± 3.0 (27–40)	11.9 ± 0.8 (11.3–15.0)
		LSR	LfL	61	27.3 ± 5.3 (20–39)	16.3 ± 4.1 (10.9–30.0)
		SSR	LfS	47	22.6 ± 2.4 (17–29)	20.2 ± 2.9 (15.5–29.5)

Abbreviations of morphs are DR, densely rakered whitefish; LSR, large sparsely rakered whitefish; SSR, small sparsely rakered whitefish. Code is a combination of lake and morph name.

**Table 3:** Summary trait table of the three morphs in each watercourse indicating mean number of gill rakers and total length (cm) of fish ± standard deviation (*SD*). Statistical significance of traits among subpopulations within lakes on different watercourses is indicated with asterisks

Watercourse	Morph	Mean number of gill rakers ± *SD*	DR	LSR	Mean length ± *SD*	DR	LSR
Alta	DR	37.4 ± 3.5			22.8 ± 2.5		
	LSR	25.7 ± 2.5	***		22.9 ± 5.7	—	
	SSR	23.4 ± 2.6	***	***/—	22.2 ± 4.8	—	—
Tana	DR	37.2 ± 3.4			18.0 ± 4.7		
	LSR	23.4 ± 3.1	***		22.2 ± 6.7	***/—	
	SSR	22.7 ± 2.1	***	*/—	22.9 ± 5.7	***/—	***/—
Pasvik	DR	34.6 ± 3.0			12.2 ± 2.7		
	LSR	25.6 ± 4.7	***		19.4 ± 6.5	***	
	SSR	20.7 ± 2.7	***	***	19.1 ± 3.4	***/—	***/**

***p < .001, **p < .01, *p < .05, — N.S.

### 3.2 Population‐to‐population coancestry matrix

**Figure 2:**
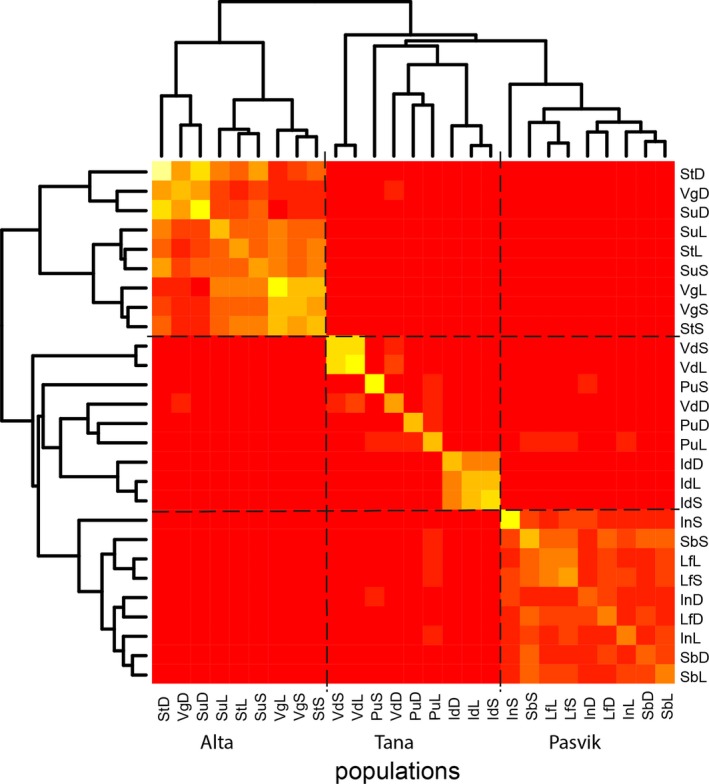
Heat map illustration of the coancestry matrix $\theta^{P}$. Watercourses are separated with hatched lines. The lighter the color is, the more the populations resemble each other. Diagonal elements of matrix $\theta_{\mathrm{ii}}^{P}$ represent random genetic drift. Dendrogram on the side illustrates the structure and hierarchical clustering of the genetic matrix. For lake and morph codes, see Table 2

The population‐level coancestry coefficient matrix $\theta^{P}$ illustrates the relatedness between populations (Table S2). Mean estimates of diagonal elements in the coancestry matrix ($\theta_{\mathrm{ii}}^{P}$) were greater in Alta and Tana than in Pasvik, suggesting that the whitefish morphs in Alta and Tana have been subjected to more genetic drift than the whitefish morphs in the Pasvik watercourse. The off‐diagonal elements of the coancestry coefficients ($\theta_{\mathrm{ij}}^{P}$) represent the interpopulation coancestry and the gene flow between populations. The level of relatedness between morphs was largest in Alta, where especially DR whitefish in different lakes clustered together and displayed high interpopulation coancestry among them, whereas relatedness between DR and the benthic morphs was smaller (Figure 2). When comparing the level of coancestry between lakes and between habitats, the differences between the three watercourses were prominent. In Alta, the average coancestry within morphs across lakes was larger than the average coancestry within lakes among morphs (Table S2). In other words, relatedness between morphs across lakes was larger than relatedness of different morphs within same lakes. Thus, the population structure was more dependent on the effect of habitat than the effect of the lake. Tana was characterized by low levels of gene flow between lakes, although the coancestry coefficient revealed low levels of relatedness between Tana, Alta, and Pasvik watercourses. However, all except one of these off‐diagonal terms of relatedness between different watercourses were ≤ 0.01 (between PuL and SbS: 0.02), which can refer to numerical noise from the MCMC calculation (Table S2). The most diverged morphs with the smallest interpopulation coancestry within lakes were in Pasvik. Although the morphs were related also across the lakes, the average relatedness within lakes was slightly greater than the average relatedness among morphs in different lakes.

### 3.3 Observed divergence versus expected divergence under drift

**Figure 3:**
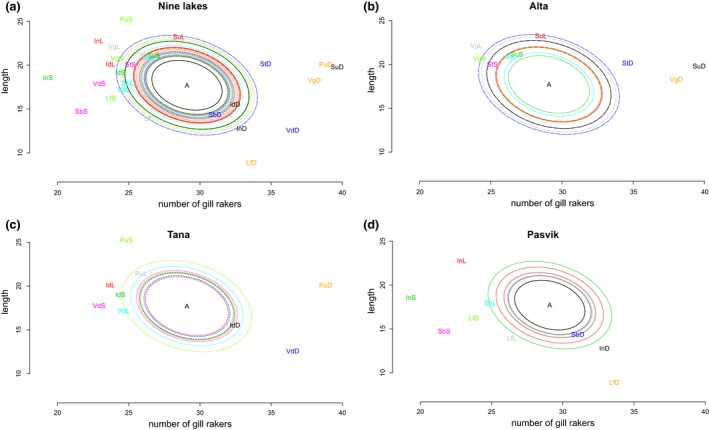
Observed divergence of phenotypic traits in all study lakes. Each ellipse represents the drift distance for the population of same color. Observed divergence in study lakes and populations (see abbreviations from Table 2). The position of the population codes represents population‐specific level of additive genetic effects, population means. The mean phenotype of each population is plotted together with estimated ancestral mean (A) and expected divergence under random genetic drift (ellipses). Each ellipse represents the drift distance for the population of the same color. The ellipses have different sizes, because the local populations experience different amounts of random genetic drift. Populations with mean value outside of their ellipses indicate divergent selection whereas populations with mean value inside the ellipse are expected to differentiate from ancestral population as a consequence of random drift

**Figure 4:**
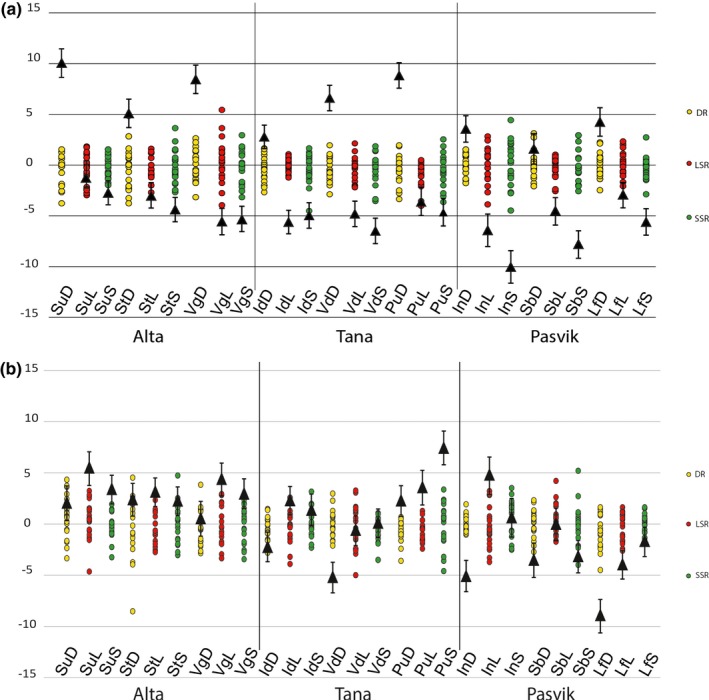
Population means in gill raker (a) and total length (b) traits. Triangular dots with error bars represent population means from the real data with 95% credibility interval. Small dots represent the amount of variation expected under drift‐based divergence. Zero level is the ancestral mean. In this picture, there are 20 simulated replicates representing different scenarios (see morph and lake abbreviations from Table 2).

Neutral divergence in phenotypic traits was assessed using the ancestral mean value and the expected divergence under random genetic drift and then compared to observed divergence on phenotypic traits (Figure 3). Signals of selection were detected for most populations and were most pronounced for the DR and the SSR whitefish (Figure 3a–d). Because the trait visualization in Driftsel is a summary statistic that combines the two traits, we also examined the estimates of population means from real data (population effect) against the ancestral mean and neutral divergence for both traits separately to gain a more detailed impression of these two traits (Figure 4). When comparing the population effect for gill raker number, the DR whitefish in all the watercourses, as well as SSR whitefish in Pasvik watercourse, showed substantial differences from the ancestral mean and drift‐based estimates (Figure 4a). Thus, diversifying selection appears to have more effect than random genetic drift on the number of gill rakers in these two morphs. In Tana and Alta, the difference between the observed population effect and the drift‐based divergence in the SSR morph was a bit smaller (Figure 4a). Although the SSR morph was further from the ancestral mean in most of lakes in these two watercourses than what would have been expected without selection, the SSR morph in Suohpatjavri, Iddjajavri, and Pulmankijärvi was still close to the neutral drift‐based divergence. When assessing the length of fish, the effect of population was not as clear as with the gill raker number (Figure 4b). The population means were not very far from the ancestral mean and were also close to the estimated drift‐based divergence. Also, here the DR whitefish showed evident separation from the ancestral mean, although the length of fish had a smaller population effect for the DR whitefish compared to the other morphs. This was most clear in the Pasvik watercourse, where the population mean for DR whitefish was moved further away from the ancestral mean than expected based on the drift alone, and DR whitefish were smaller in size than the other morphs (Figure 4b). In Alta and Tana watercourses, the populations were (with some exceptions) in general closer to the neutral drift‐based estimates. In other words, the divergence in phenotypic traits was not as pronounced in Alta and Tana watercourses as observed in the Pasvik watercourse.

In order to evaluate the effect of selection versus drift on single traits, we estimated *S* and *H* test values separately for both traits. The *S* test values for both traits were >0.95 in all three watercourses, which confirms that the differences in population means of the traits have been caused by selection as the observed diversification cannot be achieved by random genetic drift alone. The signal of selection was clear when adding habitat information to the statistical test as the *H* test values were >0.99. One exception was the *H* test –value (0.97) of fish length in Alta watercourse. The high values (>0.95) observed in the *H* test in all watercourses confirmed that there is a strong correlation between the phenotypic traits and the environment.

We found a small antagonistic relationship between number of gill rakers and length, suggesting that a higher number of gill rakers tend to be related with smaller size of fish (Figure 4). This relationship was least visible in Alta, where there were no significant differences in mean length between the whitefish morphs (Figure 4). Evaluation of the fixed effects revealed size difference between sexes, where males were slightly smaller (mean 20.2 ± 5.6 cm [*SD*]) than females (mean 20.8 ± 5.9 cm). This difference was small and statistically significant only for the LSR whitefish in Suohpatjavri and for DR and SSR whitefish in Inarijärvi. Immature fish were also slightly smaller (mean 18.9 ± 5.4 cm) than mature fish (mean 21.5 ± 5.9 cm; *p* < .001).

## 4 DISCUSSION

In the present study, we tested whether extrinsic factors repeatedly have shaped the phenotypic specializations, number of gill rakers, and fish length, in three sympatric whitefish morphs. Using a landscape‐wide study design, we also tested whether the phenotypic specializations were driven by parallel evolutionary processes. The results of our study show that phenotypic differentiation in the three whitefish morphs was a response to diversifying selection, as neutral drift‐based divergence was not able to explain the observed pattern. We observed parallel phenotype‐environment association especially in number of gill rakers among the whitefish morphs across the lakes of three watercourses.

Our results are in line with the theory of ecological speciation, which predicts that reproductive isolation evolves between populations as a by‐product of ecologically based diversifying selection (Nosil, 2012). Diversifying selection mediates the development of adaptive phenotypic traits, such as gill rakers and body size, allowing for more efficient niche utilization (Schluter, 2000). Resource competition and subsequent adaptation to a specific niche are suggested to be driving mechanisms in this divergence process (Pfennig & Pfennig, 2010), but only a few empirical studies have investigated how diversifying selection contributes to the divergence at the early stages of ecological speciation (see Bolnick & Fitzpatrick, 2007; Meyer & Kautt, 2014 for review). This also applies for the most studied examples of ecological speciation, the very diverse cichlids assemblages in tropical lakes (Malinsky et al., 2015; Seehausen, 2006), the pelagic‐benthic threespine sticklebacks (*Gasterosteus aculeatus*; Arnegard et al., 2014; McKinnon & Rundle, 2002), and Arctic charr (*Salvelinus alpinus*; Klemetsen, 2010; Recknagel, Hooker, Adams, & Elmer, 2017; Snorrason et al., 1994) in postglacial lakes. The existence of the three Fennoscandian whitefish morphs has been hypothesized to be the outcome of incipient ecological speciation toward three main lake habitats (e.g., Harrod et al., 2010; Østbye, Næsje, et al., 2005; Præbel, Knudsen, et al., 2013; Siwertsson et al., 2010), where the numbers of gill rakers and body size of the fish have been proposed as potential key adaptive traits (Kahilainen & Østbye, 2006; Præbel, Knudsen, et al., 2013). The repeated occurrence of similar morph types throughout the northern Fennoscandia has also been suggested to be the product of parallel evolution (Østbye et al., 2006). This implies that these key traits repeatedly have been subjected to diversifying selection, as it is not likely that the traits would have repeatedly evolved by random drift alone. However, no other study has addressed the effect of random drift versus selection at a landscape level (but for lake level, see Præbel, Knudsen, et al., 2013).

Morphological adaptation to a specific niche has been investigated in a range of species, for example, head morphology in ecomorphs of Arctic charr (Adams et al., 1998; Recknagel et al., 2017). In European whitefish, the divergence in number and the length of gill rakers, head morphology, and body shape have been extensively studied (e.g., Amundsen et al., 2004; Hudson, Lundsgaard‐Hansen, Lucek, Vonlanthen, & Seehausen, 2017; Kahilainen & Østbye, 2006; Siwertsson, Knudsen, Adams, et al., 2013; Siwertsson, Knudsen, Præbel, et al., 2013), where number and the length of gill rakers have been identified as one of the most important niche‐related morphological adaptations (Roesch, Lundsgaard‐Hansen, Vonlanthen, Taverna, & Seehausen, 2013). Morphological differences in gill rakers affect the foraging efficiency (Roesch et al., 2013; Sanderson, Cheer, Goodrich, Graziano, & Callan, 2001) and have shown to be correlated with habitat choice and/or prey selectivity in a range of fish species, such as cichlids (Muschick et al., 2014), alewives (*Alosa pseudoharengus*; Post, Palkovacs, Schielke, & Dodson, 2008), and sticklebacks (Schluter & McPhail, 1992). However, the mechanism behind this phenotype‐environment association is still not completely understood. In European whitefish from subarctic lakes, the number of gill rakers is positively correlated with the use of the pelagic habitat and the proportion of zooplankton in the diet (Kahilainen et al., 2011). As resource availability and prey size vary between the three principal habitats (Hayden, Harrod, & Kahilainen, 2014), similar habitats appear to produce phenotypes that are adapted to utilize habitat specific resources. In the pelagic habitat, the higher number of gill rakers facilitates consumption of small zooplankton (Roesch et al., 2013), whereas such gill rakers are not efficient for foraging larger benthic prey buried in sediment or sand (Lundsgaard‐Hansen, Matthews, Vonlanthen, Taverna, & Seehausen, 2013). In order to enable indigestible particles to exit, the feeding of large benthic prey is correlated with a smaller number of short and robust gill rakers (Kahilainen et al., 2011). Accordantly, we observed statistically significant differences in the number of gill rakers between whitefish morphs increasing from profundal to littoral to pelagic morphs in all watercourses, where the observed mean number of gill rakers correlated with the niche use observed in other studies of whitefish (e.g., Amundsen et al., 2004; Harrod et al., 2010). Similar pattern of diversifying selection between niche uses is also found among other fish species, for example, Arctic charr and three‐spined sticklebacks, in postglacial lakes (Skúlason, Snorrason, Ota, & Noakes, 1993; Taylor, 1999). Previous studies of lake whitefish and European whitefish species have revealed deviation from neutral expectation, suggesting that the number of gill rakers in whitefish has evolved as a product of natural selection (Østbye, Næsje, et al., 2005; Præbel, Knudsen, et al., 2013; Rogers et al., 2002). Our study confirms and expands these findings showing that natural selection induces distinct number of gill rakers in littoral, pelagic, and profundal morphs of European whitefish regardless of the repeated founder events. This was evident, as the observed divergence in this trait among habitats consistently was larger than the expected divergence under random genetic drift. There was furthermore consistent sign of a colonization history ranging from east to west in the ecological, phenotypic, and genetic divergence, especially toward the profundal habitat. The profundal SSR morph showed the most pronounced differences in body shape, number of gill rakers, habitat, and diet from the other benthic morph, the littoral LSR whitefish, in the oldest Pasvik watercourse populations compared to younger Alta populations (Harrod et al., 2010; Kahilainen et al., 2003; Siwertsson, Knudsen, Præbel, et al., 2013). This was also in line with the observed genetic differentiation, where the level of reproductive isolation between the littoral and profundal whitefish morphs closely followed the ecological and phenotypic divergence (this study, Præbel, Knudsen, et al., 2013; Siwertsson, Knudsen, Præbel, et al., 2013) and suggests still ongoing ecological driven divergence in the westernmost Alta watercourse. In our study, the SSR whitefish in Alta and Tana watercourses showed significant signs of selection, although the population mean in number of gill rakers was not very far from the expected drift‐based divergence. This may be due to shorter evolutionary time for divergence, differences in selection pressure between watercourses (e.g., lake depth, prey resources, and predation pressure), and/or the populations′ potential to respond to selection (Nosil, Harmon, & Seehausen, 2009).

We observed a small antagonistic relationship between gill raker number and total length of fish. Feeding on zooplankton is usually associated with larger number of gill rakers, small body size, and slower growth as an effect of lower energy content and high population density, compared to benthic feeders (Kahilainen et al., 2003, 2005; Link & Hoff, 1998). Small body size and early sexual maturation of DR whitefish are also likely life‐history adaptations to high predation‐induced mortality, as this morph is the main prey for piscivorous fish such as pelagic brown trout (Jensen et al., 2008; Kahilainen & Lehtonen, 2003). For the other morphs, predation mortality is much lower: LSR whitefish is able to reach a size refuge from the gape size of piscivorous fish (Bøhn, Amundsen, Popova, Reshetnikov, & Staldvik, 2002), whereas SSR whitefish utilizes the dark profundal habitat with very low amount of predators (Kahilainen & Lehtonen, 2003). However, predation‐induced mortality is likely dependent on the abundance of piscivorous fish. In the Pasvik and Tana lakes with abundant and diverse predator populations, the DR whitefish were indeed smaller in size than the other two morphs. On the contrary, there were no significant differences in fish length between morphs in Alta. However, the mean body size of populations is dependent not just on the genes each individual possesses but also on environmental aspects such as prey availability and density‐dependent resource competition (Kahilainen et al., 2003, 2005; Muir et al., 2010). As the heritability estimates for length are smaller than for the number of gill rakers, we have to take into account that phenotypic plasticity may have a larger effect on the length than on the number of gill rakers.

The buildup of reproductive isolation plays an important role in the speciation process. Populations that experience ecologically based diversifying selection are also subjected to diminishing amount of gene flow between them, which leads to development of reproductive isolation. One approach to assess how far the speciation process has proceeded is to measure the reproductive isolation between populations, as multiple studies have detected association between reproductive isolation and ecological divergence (e.g., Funk, Nosil, & Etges, 2006; Hendry, 2004; Lu & Bernatchez, 1999). An examination of the coancestry matrix obtained herein revealed different scenarios of relatedness and population structure between the three watercourses. In the Tana watercourse, all the study lakes appeared to form their own cluster, and there was little relatedness between the three study lakes that were located far apart. In the Pasvik watercourse, relatedness between whitefish populations was in general smaller than in the Alta watercourse, where especially the DR whitefish clustered together and had high interpopulation coancestry among them. The whitefish populations shared some level of genetic similarity, not just among morphs (across lakes), but also within different lakes. This may refer to parallel divergence in the same direction across lakes, even when the reproductive isolation between morphs is weak (see also Johannesson, 2001).

The observed phenotypic diversification in gill raker number has been proposed to have a recent origin (Østbye, Bernatchez, et al., 2005). Rapid response to selection is possible when the trait has a strong (additive) genetic basis and enough genetic variation at the initial stages of divergence (Hirsch, Eckmann, Oppelt, & Behrmann‐Godel, 2013; Kopp & Matuszewski, 2014). Selection pressure and gene flow between subpopulations have effect on how fast the divergence builds up (Hendry, Wenburg, Bentzen, Volk, & Quinn, 2000). If there is substantial amount of gene flow between populations, the divergence may not arise as gene flow works against segregation, especially in a case of multifarious selection, where selection is operating on multiple genetically independent traits (Nosil, Harmon, et al., 2009). Gene flow may therefore slow down or prevent local adaptation and the formation of discrete populations (Blanquart, Gandon, & Nuismer, 2012; Nagylaki & Lou, 2008; Slatkin, 1985). Drift may contribute to the divergence even under conditions of strong natural selection. When the level of gene flow between populations is high, drift may overrun the joint effect of selection and gene flow, as selection and migration may reverse each other's effect (Savolainen, Lascoux, & Merilä, 2013). In our study, the phenotypically and genetically most divergent whitefish populations were found in the oldest Pasvik watercourse, whereas the whitefish populations in the Alta watercourse were more influenced by drift due to founder effects. Notwithstanding, the three morphs have still evolved in all three watercourses, but the radiations appear to be at different stages of divergence. As the divergence likely have happened repeatedly in each lake (Østbye et al., 2006), “the starting gene pool” for the divergence process has most likely not been the same among watercourses. Nevertheless, we detected signals of selection also in the younger whitefish populations in the Alta watercourse. Labonne et al. (2016) demonstrated how selection may actively work to increase genetic variation, even in a case of strong founder effect and minimal genetic variation. Thus, reduced genetic variation does not necessarily prevent adaptation, as small populations may still retain their adaptive potential (Wood, Tezel, Joyal, & Fraser, 2015). However, when selection is operating with smaller degree of genetic variation, it may require a longer evolutionary time for niche differentiation, adaptation, and ecological speciation (Gavrilets, 2004; Hendry, 2009). Although the pelagic DR whitefish is found across all three watercourses, the substantially diverged SSR whitefish is currently present only in the older watercourses. Previous studies have proposed the existence of distinct European whitefish morphs as a result of parallel evolution (Østbye et al., 2006; Præbel, Knudsen, et al., 2013; Siwertsson, Knudsen, Adams, et al., 2013). The existence of a single mtDNA lineage and genetic clustering of morphs in their respective lakes or watercourses gives support to this hypothesis (Østbye, Bernatchez, et al., 2005). The possibility of neutral divergence due to random genetic drift is considered as an unlikely explanation for the repeated occurrence of phenotypically differentiated morphs in the three main habitats in many lakes across Fennoscandia. However, no previous studies have tested this hypothesis using a large landscape level dataset as in the current study. We observed parallel pattern of divergence across lakes, while the degree of divergence varied between watercourses. When assessing lakes separately, with different amount of standing genetic variation, it is likely that selection has been working with a different set of genetic variation in each lake.

To conclude, we have presented new results on the interplay between diversifying selection and random genetic drift in the evolution of local adaptation. Our results show that natural selection has worked toward stronger phenotype‐environment correlations for the size of the fish and the number of gill rakers, where especially the gill raker number of a whitefish morph is an adaptation toward a more efficient use of the specific lake habitat. Further studies are necessary to understand the genetic mechanisms behind the diversification, and to what extent and how traits evolve at different levels of standing genetic variation.

## CONFLICT OF INTEREST

None declared.

## AUTHOR CONTRIBUTIONS

KP conceived the study; KH, KØ, and KP designed the study; KH, KKK, KP, and P‐AA collected the samples; KH and KP performed the analysis; KH drafted the article; and all authors contributed critically to the revisions and gave final approval for publication.
