# Supplementary material for: Diversifying selection drives parallel evolution of gill raker number and body size along the speciation continuum of European whitefish
Source: Ecol Evol. 2018 Feb 5;8(5):2617–31. doi: 10.1002/ece3.3876 (PMC5838045; doi:10.1002/ece3.3876)
Supplement: Supplementary file 1 [file ECE3-8-2617-s001.docx]

|  |  |  |  |  |  |  |  |  |
| --- | --- | --- | --- | --- | --- | --- | --- | --- |

Supplementary material

Table S1. Details of the microsatellite loci, total number of alleles in each locus, and number of alleles in each locus per population. Bolded microsatellite markers were used in this study.

|  |  | Number of allele per population | | | | | | | | | | | | | |  |  | |  | |  | |  | |  | |  | |  | |  | |  | |  | |  | |  | |  |
| --- | --- | --- | --- | --- | --- | --- | --- | --- | --- | --- | --- | --- | --- | --- | --- | --- | --- | --- | --- | --- | --- | --- | --- | --- | --- | --- | --- | --- | --- | --- | --- | --- | --- | --- | --- | --- | --- | --- | --- | --- | --- |
| Locus ID | Total | SuD | SuL | SuS | StD | StL | StS | VgD | VgL | VgS | IdD | IdL | IdS | VdD | VdL | | VdS | PuD | | PuL | | PuS | | InD | | InL | | InS | | SbD | | SbL | | SbS | | LfD | | LfL | | LfS | |
| BWF1^a^ | 12 | 3 | 4 | 3 | 2 | 3 | 4 | 3 | 6 | 4 | 5 | 5 | 5 | 6 | 6 | | 6 | 9 | | 6 | | 6 | | 6 | | 6 | | 6 | | 7 | | 6 | | 7 | | 7 | | 7 | | 7 | |
| BWF2^a^ | 9 | 2 | 2 | 4 | 3 | 3 | 3 | 2 | 2 | 4 | 3 | 4 | 3 | 4 | 4 | | 5 | 5 | | 5 | | 4 | | 6 | | 6 | | 4 | | 7 | | 5 | | 7 | | 6 | | 7 | | 6 | |
| BFRO-018^b^ | 10 | 1 | 1 | 1 | 1 | 1 | 1 | 1 | 1 | 1 | 4 | 6 | 2 | 1 | 2 | | 2 | 4 | | 4 | | 4 | | 4 | | 3 | | 2 | | 3 | | 5 | | 4 | | 4 | | 6 | | 6 | |
| **ClaTet1**^c^ | 20 | 4 | 6 | 5 | 6 | 4 | 5 | 5 | 7 | 5 | 10 | 8 | 5 | 10 | 7 | | 7 | 11 | | 9 | | 6 | | 12 | | 12 | | 8 | | 13 | | 16 | | 13 | | 15 | | 13 | | 12 | |
| **ClaTet3**^c^ | 20 | 4 | 4 | 3 | 5 | 5 | 6 | 5 | 4 | 4 | 8 | 9 | 7 | 7 | 8 | | 8 | 13 | | 7 | | 8 | | 8 | | 10 | | 7 | | 7 | | 11 | | 10 | | 7 | | 12 | | 9 | |
| ClaTet06^c^ | 30 | 5 | 7 | 4 | 6 | 6 | 6 | 4 | 8 | 7 | 10 | 13 | 8 | 13 | 14 | | 11 | 17 | | 12 | | 12 | | 16 | | 13 | | 9 | | 13 | | 17 | | 15 | | 16 | | 17 | | 15 | |
| **ClaTet09**^c^ | 16 | 7 | 8 | 6 | 7 | 8 | 4 | 7 | 9 | 7 | 9 | 11 | 6 | 11 | 7 | | 8 | 13 | | 11 | | 8 | | 13 | | 7 | | 8 | | 12 | | 13 | | 13 | | 11 | | 13 | | 10 | |
| ClaTet10^c^ | 39 | - | - | - | - | - | - | - | - | - | 21 | 25 | 17 | - | - | | - | 22 | | 18 | | - | | - | | - | | - | | - | | - | | - | | - | | - | | - | |
| ClaTet05 ^c^ | 9 | - | - | - | - | - | - | - | - | - | - | - | - | - | - | | - | 7 | | 9 | | - | | - | | - | | - | | - | | - | | - | | - | | - | | - | |
| **ClaTet13**^c^ | 12 | 6 | 6 | 6 | 7 | 6 | 7 | 7 | 6 | 6 | 8 | 8 | 6 | 7 | 7 | | 7 | 7 | | 7 | | 7 | | 8 | | 6 | | 5 | | 9 | | 7 | | 7 | | 8 | | 10 | | 10 | |
| **ClaTet15**^c^ | 8 | 4 | 4 | 3 | 5 | 5 | 4 | 3 | 5 | 5 | 4 | 4 | 4 | 4 | 3 | | 3 | 5 | | 4 | | 3 | | 6 | | 5 | | 3 | | 4 | | 4 | | 5 | | 4 | | 6 | | 4 | |
| **ClaTet17**^c^ | 32 | 8 | 6 | 5 | 7 | 8 | 7 | 8 | 9 | 9 | 20 | 18 | 15 | 16 | 16 | | 14 | 13 | | 14 | | 9 | | 14 | | 13 | | 9 | | 15 | | 14 | | 18 | | 19 | | 19 | | 16 | |
| **ClaTet18**^c^ | 15 | 2 | 2 | 2 | 3 | 2 | 3 | 3 | 3 | 3 | 4 | 4 | 3 | 3 | 3 | | 3 | 4 | | 5 | | 4 | | 6 | | 7 | | 3 | | 7 | | 5 | | 6 | | 5 | | 7 | | 7 | |
| **Cocl-Lav4**^c^ | 6 | 1 | 1 | 1 | 1 | 1 | 1 | 1 | 2 | 1 | 4 | 1 | 2 | 3 | 3 | | 2 | 4 | | 4 | | 3 | | 4 | | 4 | | 3 | | 3 | | 4 | | 5 | | 2 | | 3 | | 3 | |
| **Cocl-Lav6**^c^ | 12 | 2 | 2 | 2 | 2 | 2 | 2 | 2 | 2 | 2 | 8 | 7 | 6 | 6 | 6 | | 6 | 7 | | 6 | | 5 | | 8 | | 6 | | 5 | | 7 | | 7 | | 6 | | 7 | | 7 | | 8 | |
| Cocl-Lav10^d^ | 4 | 3 | 3 | 3 | 3 | 3 | 3 | 3 | 3 | 3 | 3 | 2 | 2 | 3 | 3 | | 3 | 3 | | 3 | | 3 | | 4 | | 4 | | 3 | | 3 | | 3 | | 3 | | 3 | | 3 | | 3 | |
| **Cocl-Lav18**^d^ | 8 | 2 | 2 | 1 | 3 | 2 | 1 | 2 | 3 | 2 | 4 | 4 | 3 | 2 | 2 | | 2 | 2 | | 4 | | 2 | | 2 | | 2 | | 2 | | 2 | | 2 | | 3 | | 2 | | 2 | | 2 | |
| **Cocl-Lav27**^d^ | 7 | 2 | 2 | 2 | 3 | 3 | 2 | 1 | 3 | 2 | 4 | 4 | 2 | 2 | 4 | | 5 | 4 | | 4 | | 2 | | 2 | | 1 | | 1 | | 2 | | 4 | | 2 | | 3 | | 4 | | 3 | |
| **Cocl-Lav49**^d^ | 16 | 4 | 3 | 3 | 4 | 3 | 4 | 3 | 3 | 3 | 5 | 4 | 5 | 12 | 6 | | 6 | 5 | | 5 | | 6 | | 6 | | 7 | | 7 | | 6 | | 6 | | 8 | | 7 | | 7 | | 7 | |
| **Cocl-Lav52**^d^ | 36 | 7 | 6 | 2 | 9 | 7 | 9 | 6 | 8 | 6 | 12 | 13 | 8 | 12 | 17 | | 13 | 11 | | 9 | | 7 | | 15 | | 9 | | 11 | | 10 | | 12 | | 13 | | 14 | | 15 | | 17 | |
| C2-157^e^ | 20 | 1 | 2 | 1 | 3 | 4 | 1 | 2 | 3 | 4 | 11 | 9 | 8 | 7 | 6 | | 6 | 6 | | 7 | | 7 | | 8 | | 7 | | 7 | | 7 | | 10 | | 10 | | 8 | | 10 | | 9 | |
|  |  |  |  |  |  |  |  |  |  |  |  |  |  |  | |  |  | |  | |  | |  | |  | |  | |  | |  | |  | |  | |  | |  | |  |

^a^(Patton et al., 1997); ^b^(Susnik et al., 1999);  ^c^ (Winkler & Weiss, 2008); ^d^ (Rogers et al., 2004); ^e^(Turgeon et al., 1999)
